# Identifying obesity indicators which best correlate with type 2 diabetes in a Chinese population

**DOI:** 10.1186/1471-2458-12-732

**Published:** 2012-09-01

**Authors:** Zhong Xin, Chang Liu, Wen-Yan Niu, Jian-Ping Feng, Lei Zhao, Ya-Hong Ma, Lin Hua, Jin-Kui Yang

**Affiliations:** 1 Department of Endocrinology, Beijing Tongren Hospital, Capital Medical University, 1 Dong Jiao Min Xiang, Beijing 100730, China; 2 Department of Immunology, Key Laboratory of Immuno Microenvironment and Disease of the Educational Ministry of China, Tianjin Medical University, Tianjin, 300070, China; 3 Department of Endocrinology, the PLA 305 Hospital, Beijing, 100017, P. R. China; 4 Department of Mathematics, School of Biomedical Engineering, Capital Medical University, Beijing, 100069, China

**Keywords:** Type 2 diabetes, Central obesity, Waist circumference, BMI

## Abstract

**Background:**

Obesity has been shown to be a prognostic indicator of type 2 diabetes (T2D); however, the power of different obesity indicators in the detection of T2D remains controversial. This study evaluates the detecting power of body mass index (BMI), waist circumference (WC), waist-to-hip ratio (WHR) and waist-to-height ratio (WHTR) for the presence of T2D in undiagnosed diabetics among the Chinese population.

**Methods:**

Individuals were selected from an ongoing large-scale population-based Beijing Community Pre-Diabetes (BCPD) study cohort. The oral glucose tolerance tests (OGTT) were performed to diagnose diabetes. A total of 220 new cases of T2D and 1,868 normal blood glucose subjects were analyzed. ROC curve analyses were used to compare the association of different obesity indicators with T2D and determine the optimal cut-off points of the best predictor for identifying T2D in men and women.

**Results:**

All indicators positively correlated with presence of T2D in both men and women. In women, WC, WHR and WHTR were similar, but were better in identifying T2D when compared to BMI (P < 0.0001, P=0.0016 and P=0.0001, respectively). In men, WC, WHTR and BMI were similar, but WC and WHTR were better than WHR (P=0.0234, P=0.0101, respectively). For women, 86 cm was the optimal WC cut-off point, and its sensitivity and specificity were 0.714 and 0.616; for men, the optimal cut-off point was 90 cm, and its sensitivity and specificity were 0.722 and 0.571.

**Conclusion:**

Compared with BMI, WHR and WHTR, WC is a simple and accurate measure for predicting T2D in the Chinese population.

## Background

Obesity has become a public health problem in both developed and developing countries [1]. As its prevalence increases, so do the risks of comorbidities, including the risk for type 2 diabetes (T2D). In an attempt to curtail long term costs of managing these disease states, prevention and control have become key aspects of prophylaxis and treatment [2]; however, identifying those parameters that strongly correlate to obesity and associated comorbidities remain controversial, specifically those markers for diabetes.

At present, the independent effects of abdominal adiposity compared with overall obesity are not well characterized [2,3]. Although it has been shown that waist circumference (WC) is a better predictor of diabetes than body mass index (BMI) [4,5], the data are often inconclusive [6,7] and the use of WC and waist-to-hip ratio (WHR) remains controversial [3,8,9]. Additional parameters, including waist-to-height ratio (WHTR) have also been evaluated for predictive value and has demonstrated a positive correlation for diabetes and metabolic risk in Asian populations [10].

The rising cost of treating diabetes and its complications is a major concern in China [11,12]. Currently, it is believed there is large population with undetected T2D in rural areas and the use of simple screening, such as anthropometric markers, could be used in the detection and management of the disease [11]. To date, few studies have been published identifying which obesity indicator and its optimal cutoff points (sensitivity and specificity) are the best predictors of T2D in Chinese populations. Recently, Bao et al. reported cutoff points for waist circumference and their predictive value for abdominal obesity and risk of metabolic syndrome (MetS) in the Chinese population [13]. However, the independent predictive power of MetS in predicting the risk of cardiovascular diseases may not be stronger than its single components [14,15]. Therefore, we compared the performance of BMI, WC, WHR and WHTR to diagnosed T2D and evaluated optimal cutoff points using undiagnosed diabetes and oral glucose tolerance test (OGTT), to assess diabetes risk in Chinese.

## Methods

### Subjects

Individuals were selected from an ongoing large-scale population-based cohort (the Beijing Community Pre-Diabetes (BCPD) study). This study is designed to facilitate the conduct of genetic epidemiology investigations and clinical trials, individuals originated from the settled community of Nanfaxin, a satellite rural town of Beijing [16]. Participants without previously known diabetes were selected from the 2826 registered individuals aged ≥ 35 years in the year 2007. All subjects were invited to attend a baseline examinations including anthropometric and blood pressure measurements, in addition to completing a general health questionnaire. Subjects whose fasting plasma glucose (FPG) was ≥ 5.6 mmol/L performed a 75 g oral glucose tolerance test [17]. According to the 1999 World Health Organization criteria, a total of 220 new cases of T2D and 1,868 normal blood glucose subjects were identified for the study.

The study was conducted with the approval from the Ethics Committee of Beijing Tongren Hospital, Capital Medical University. Written informed consent was obtained from each participant.

### Anthropometric measurements

Participants’ body weight (kg) and height (cm) were recorded. Height was measured with a stadiometer to the nearest 0.5 cm. Body weight was measured on a calibrated balance scale. WC and hip circumference were obtained using a cloth tape. The WC was defined as the midpoint between the peak of the iliac crest and the nadir of the costal margin in the midaxillary line. The hip circumference was measured at the level of the greater femoral trochanters [5]. These measurements were used to compute the waist circumference divided by the hip circumference [waist-hip ratio (WHR)] and waist circumference divided by the height (WHTR). The BMI was calculated by dividing the weight in kilograms by the square of the height in meters.

### Statistical analysis

The area under the receiver-operating characteristics curves (AUC) was calculated for each index of obesity and T2D using receiver-operating characteristic (ROC) curves. ROC curve is a graphic representation of the relationship between sensitivity and specificity for a diagnostic test. It provides a simple tool for comparing the predictive power of different tests or measures. ROC curves are drawn by plotting the sensitivity (true-positive rate) against the false-positive rate (1-specificity) for several measures or choices. The measure with a ROC curve that is closest to the upper left corner has the highest sensitivity and specificity and, thus, is the best predictive measure [7]. ROC curve analyses and the respective AUC were used to compare the association of WC, WHR, WHTR and BMI with T2D. The general approach to assessing whether the difference in the areas under two ROC curves is to calculate generalized Z-statistics [18], which is described as following:

(1) $$Z=\frac{A_{1}-A_{2}}{\sqrt{{\left[SE\left(A_{1}\right)\right]}^{2}{\left[SE\left(A_{2}\right)\right]}^{2}-2r\left[SE\left(A_{1}\right)\right]\left[SE\left(A_{2}\right)\right]}}$$

where A_i_ and SE(A_i_) (i=1,2) indicate the observed area and estimated standard error of A_i_; and r represents the estimated correlation between A_1_ and A_2_. This quantity z is the approximate normal distribution, and can be used to compare the AUCs. ROC curves were also used to calculate the sensitivity, specificity and Youden’s index, defined as “sensitivity + specificity-1”. These determined the optimal values for each predictor of T2D. All statistical analyses were conducted with the software package SPSS version 11.5 (SPSS Inc., Chicago, IL, USA) for Windows and MedCalc version 11.4 (http://www.medcalc.be)*.*

Consider the major statistic methods used in this paper were the comparison of AUCs, we calculated the sample size using ROCPWR software (http://metz-roc.uchicago.edu/), which can compute the statistical power by using the estimates of the parameters involved in the statistical tests [19]. According to the criterion of alpha=0.05, 220 T2D cases and 1,868 controls can make the statistical power reach to 0.872. Therefore, these selected samples can support our analysis and conclusion.

## Results

Table1 identifies all study cohort characteristics. Patients (men and women) with T2D rated significantly higher in weight, WC, hip circumference, BMI, WHR and WHTR, compared with subjects with normal blood glucose (p < 0.001).

**Table 1:** Characteristics of study cohorts

	**Normal**	**T2D**	**P-value**
Participants(n)	1868	220	
Age(year)	51.66 ±10.35	56.22 ±10.06	<0.001
Height(cm)			
Men	167.02 ± 6.54	166.56 ± 6.03	0.499
Women	156.68 ± 5.61	155.96 ± 5.81	0.193
Weight(Kg)			
Men	71.61 ± 11.54	77.97 ± 11.87	<0.001
Women	63.36 ± 10.11	67.50 ± 11.04	<0.001
Hip circumference (cm)			
Men	97.99 ± 7.11	102.46 ± 6.94	<0.001
Women	98.22 ± 7.84	101.08 ± 7.67	<0.001
WC (cm)			
Men	88.58 ± 9.96	95.92 ± 10.12	<0.001
Women	83.83 ± 9.92	91.33 ± 9.85	<0.001
BMI (kg/m^2^)			
Men	25.63 ± 3.62	28.05 ± 3.67	<0.001
Women	25.76 ± 3.79	27.77 ± 4.18	<0.001
WHR			
Men	0.90 ± 0.06	0.94 ± 0.06	<0.001
Women	0.85 ± 0.06	0.90 ± 0.06	<0.001
WHTR			
Men	0.53 ± 0.06	0.58 ± 0.06	<0.001
Women	0.54 ± 0.07	0.59 ± 0.07	<0.001

Unpaired t-tests were performed to determine statistical significance. All values are reported as means ± standard deviation.

*BMI*, body mass index; *WC*, waist circumference; *WHR*, waist-to-hip ratio; *WHTR*, waist-to-height ratio.

**Figure 1:**
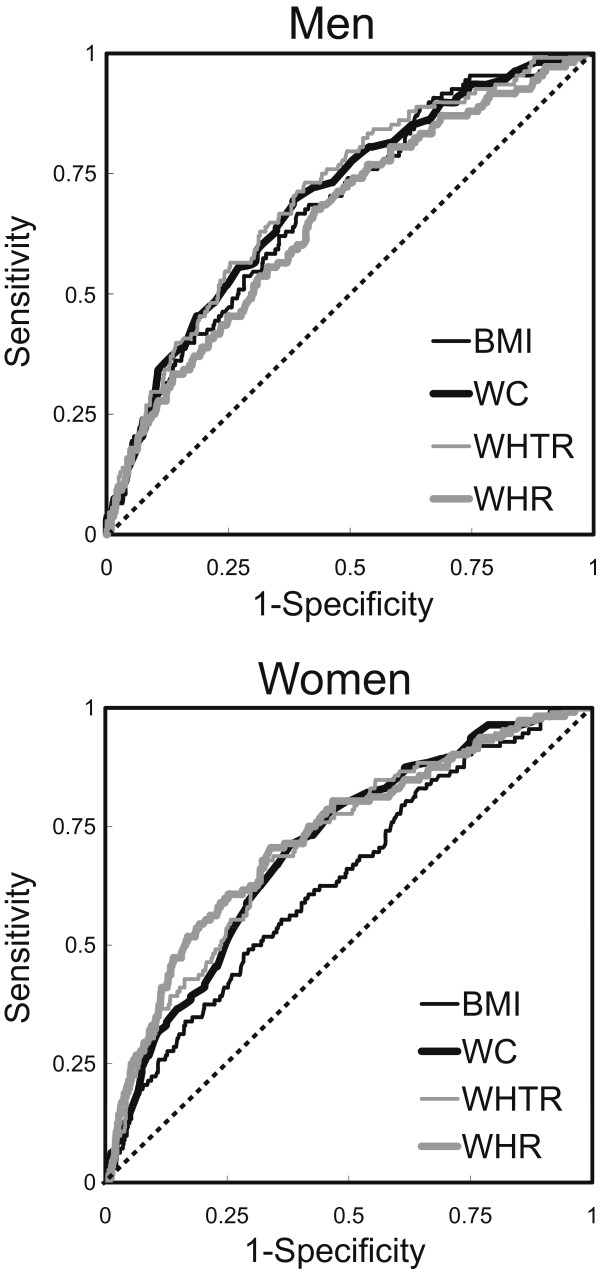
**ROC curves of different anthropometric markers in detecting T2D in men and women.** All indicators strongly correlate with T2D in both men and women (P < 0.001). BMI, body mass index; WC, waist circumference; WHR, waist-to-hip ratio; WHTR, waist-to-height ratio.

Figure1 shows the ROC curves for BMI, WC, WHR and WHTR in detecting T2D in men and women. In men, the AUC was 0.706 (95% CI: 0.655–0.759) for WHTR, 0.698 (9% CI: 0.646–0.751) for WC, 0.681 (95% CI: 0.628–0.738) for BMI and 0.656 (95% CI: 0.600–0.711) for WHR, respectively. In women, the AUC was 0.725 (95% CI: 0.674–0.776) for WHR, 0.710 (95% CI: 0.661–0.760) for WHTR, 0.706 (95% CI: 0.658–0.753) for WC and 0.636 (95% CI: 0.584–0.689) for BMI, respectively. All indicators strongly correlate with an increased risk of T2D in both men and women (P < 0.001).

To further examine the significant differences of various indicators, we compared AUC values. In men, WHTR, WC and BMI were significantly similar (P=0.2490 BMI and WC, P=0.0972 BMI and WHTR, P=0.4130 WC and WHTR); however, WC and WHTR were better indicators than WHR as determined by the AUC (P=0.0234 WC and WHR, P=0.0101 WHTR and WHR). In women, WC, WHR and WHTR were significantly similar (P=0.2685 WC and WHR, P=0.5661 WC and WHTR, P=0.3749 WHR and WHTR), and were better indicators than BMI in identifying T2D (P < 0.0001, P=0.0016, P=0.0001). These findings demonstrated that the association of WHTR and WC was higher than that for other indicators for T2D and their detecting powers are similar among genders.

We also assessed the optimal cut-off points for WC for identifying diabetes in men and women. Table2 shows the sensitivity, specificity and Youden’s index for the various cut-off points for WC in men and women. For women, 86 cm was the optimal WC cut-off point in terms of the Youden’s index, and its sensitivity and specificity were 0.714 and 0.616 respectively. For men, the optimal cut-off point for WC was 90 cm, and its sensitivity and specificity were 0.722 and 0.571 respectively.

**Table 2:** Sensitivity, specificity and Youden’s index using sex-specific cut-off points for WC to detect diabetes

**Men**			
**WC(cm)**	**Sensitivity**	**Specificity**	**Youden’s index**
84	0.861	0.337	0.198
86	0.815	0.414	0.229
88	0.778	0.495	0.273
90	0.722	0.571	0.293
92	0.639	0.650	0.289
94	0.556	0.727	0.283
**Women**			
**WC(cm)**	**Sensitivity**	**Specificity**	**Youden’s index**
80	0.875	0.384	0.259
82	0.821	0.461	0.283
84	0.777	0.543	0.320
86	0.714	0.616	0.330
88	0.625	0.682	0.307
90	0.580	0.710	0.290

The optimal cut-off point for women using the Youden’ s index is a WC of 86 cm. For men it is a WC of 90 cm. *WC* waist circumference.

## Discussion

Obesity is a recognized risk factor for numerous disease states, including T2D. Its prevalence in China, as well as the world, has become a major public health concern [20]. Although obesity is well recognized, T2D often goes undetected [11] until patients present with diabetic complications. Anthropometric measures are a simple and useful tool that can be used to screen for T2D. Although imaging techniques can accurately quantify body fat and predict metabolic abnormalities, it is impractical for routine clinical practice [21]. The Diabetes Prevention Program sub study showed that visceral fat measured by CT provided no significant advantage over simple measures in predicting the development of diabetes [5]. Therefore, the use of anthropometric measures is an alternative for rural general practitioners to assess the risks of their patients quickly, easily and inexpensively.

There has been much discussion and no clear consensus on which obesity measure is the most sensitive or specific for detecting diabetes. Previous studies comparing WC, BMI and WHR have been inconsistent [6-8] with few studies addressing these issues in the Chinese population. The aim of this study is to compare the use of these anthropometric markers in Chinese individuals and determine the best obesity indicators. Analysis of ROC curves demonstrated that WC, WHTR and BMI in men were similar, with WHR being the weakest indicator. However, in women, WC, WHR and WHTR were significantly better than BMI. These findings suggest that WC and WHTR perform significantly better than others for identifying T2D irrespective of gender. However, WC is easier to be obtained and understood than WHTR. Therefore, we chose WC to be a superior tool for discriminating obesity related T2D risk evaluation in Chinese population.

Our data among the Chinese population is supported by the literature. Wannamethee et al. showed that WC and BMI had similar predictive risks for T2D in older European men, whereas WC was a superior predictor in women [22]. Among the ethnically diverse population studied in the Diabetes Prevention Program, increased WC was the most significant predictor of diabetes in men and women in the lifestyle and placebo groups [5]. The Healthy Twin Study showed WC, WHTR, and BMI were consistently associated with all metabolic risk factors regardless of the subject’s gender in Korean population [10]. Additionally, WC is recommended by the US National Institutes of Health clinical guidelines for the assessment or management of obesity [23], and compared to WHTR, WHR and BMI, WC is not affected by measurement errors and does not require any calculations. Therefore, we recommend WC as the best obesity measure in T2D risk evaluation for the Chinese population.

The cut-off point for the WC recommended for use in clinical practice also remains controversial [24]. Different WC cutoff values are recommended for different ethnicities [25]. Several studies have examined appropriate cutoffs for abdominal obesity in Chinese population. Zhou et al’s study showed a waist circumference greater than 85 cm for men and 80 cm for women were recommended as the cut-off points for central obesity [2]. Bao Y et al’s study showed the optimal cutoff point of waist circumference that positively correlated with the risk of MetS is 90 cm for men and 85 cm for women [13]. A study found that sensitivity equaled specificity for diabetes suggested a waist circumference cutoff of 80 cm for both men and women [26]. However, a limitation of the study included patients with undiagnosed and diagnosed diabetes without performing an OGTT. Our data indicated optimal discrimination for diabetes for WC thresholds to be 86 cm in women and 90 cm in men. The influence of abdominal fatness on a health risk such as T2D is a continuous one, and thus, any cutoffs may be arbitrary [6]*.* Those recommended based on our data were identified as the values of the WC that best balanced sensitivity and specificity. This decision rule accommodates the desire to prevent a significant risk of diabetes. Our cut-off points identify risk factors with a sensitivity greater than 70% and specificity greater than 50%. It can offer an alert about the practical boundary for initiating intervention to prevent and control the increase in the risk factor of T2D as early as possible.

There are several potential limitations in this study. First, it is a cross-sectional study, and we cannot draw conclusions about cause and effect relationships between WC and T2D. Secondly, there are variations in the WC and BMI among Chinese in different regions. People living in the northern area have larger WC and BMI than those in the eastern, western and southern areas of China [27]. Our subjects were selective to the northern regions of China and therefore our results may not represent the whole population. Further research on other regions in China will be needed to identify the best cutoff of T2D and anthropometric indices. Finally, not all subjects performed OGTT. Therefore, it is possible that the frequency of T2D had been under-estimated.

## Conclusion

Our data indicates that WC is the best anthropometric measure for detecting T2D in the Chinese population. Given our evidence, the appropriate cutoffs of the WC were 90 cm in men and 86 cm in women for detecting T2D. This will help general practitioners to assess the risks of their patients quickly and easily.

## Competing interest

The authors declare that they have no competing interests.

## Authors’ contributions

Researched data, wrote manuscript, and contributed to the discussion: XZ. Researched data: LC, NWY, FJP, ZL, MYH, HL. Researched data. Designed, wrote manuscript, and contributed to the discussion: YJK. All authors read and approved the final manuscript.

## Pre-publication history

The pre-publication history for this paper can be accessed here:

http://www.biomedcentral.com/1471-2458/12/732/prepub

## Author details

^1^Department of Endocrinology, Beijing Tongren Hospital, Capital Medical University, 1 Dong Jiao Min Xiang, Beijing 100730, China. ^2^Department of Immunology, Key Laboratory of Immuno Microenvironment and Disease of the Educational Ministry of China, Tianjin Medical University, Tianjin 300070, China. ^3^Department of Endocrinology, the PLA 305 Hospital, Beijing 100017, P. R. China. ^4^Department of Mathematics, School of Biomedical Engineering, Capital Medical University, Beijing 100069, China.
